# Alkali-Soluble Resins as pH-Responsive Protective Colloids

**DOI:** 10.1021/acsami.5c12131

**Published:** 2025-07-16

**Authors:** Mehdi Naderi, Kiarash Farajzadehahary, Timo Melchin, Hans-Peter Weitzel, Jose R. Leiza, José M. Asua

**Affiliations:** † POLYMAT and Department of Applied Chemistry, Faculty of Chemistry, 652638 University of the Basque Country UPV/EHU, Joxe Mari Korta Center, Avenida Tolosa 72, 20018 Donostia-San Sebastián, Spain; ‡ 39123 Wacker Chemie AG, Johannes-Hess-Str. 24, 84489 Burghausen, Germany

**Keywords:** alkali-soluble resins, pH-responsive latexes, redispersible polymer powders, emulsion polymerization

## Abstract

Emulsion polymers are widely present in numerous applications such as adhesives, coatings, paints, and cementitious materials. In applications such as construction, they are favored in dry form that can be restabilized in the final formulation in the presence of water. This offers advantages in terms of storage, transportation, and final application. This work investigates a pH-switchable stabilizer. Turning off the stabilizer leads to latex particle coagulation and dry polymer isolation. Then, turning it back on recovers the restabilized particles in the form of an emulsion. The success of this process relies on the switchability of the colloidal stabilization system and the surface protection of low glass transition temperature (*T*_g_ < 20 °C) particles through a hard shell, ensuring particle integrity during coagulation and polymer isolation. Carboxyl-containing alkali-soluble resins are identified as promising candidates capable of providing both requirements simultaneously. Low *T*_g_ (14 °C) polymer particles of vinyl acetate and vinyl neodecanoate (VeoVa10) stabilized by high *T*_g_ alkali-soluble resins were synthesized in seeded semicontinuous emulsion polymerization. The polymer isolation was performed through a sequence of steps, including latex coagulation at pH ≤ 3, water filtration (resulting in approximately 70% water removal), drying, grinding, and reinforcement with kaolin as an anticaking agent. The isolated polymer product was then successfully restabilized in the presence of water by applying energy and increasing the pH.

## Graphical abstract

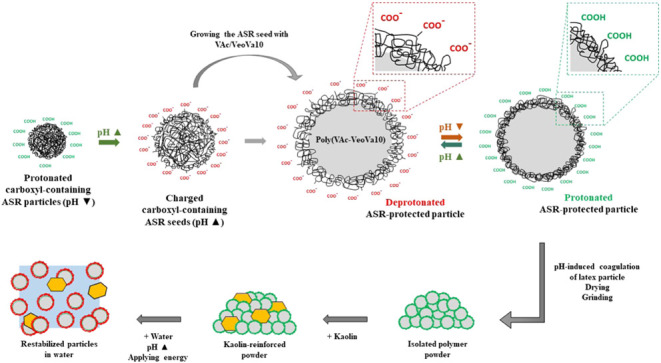

## 1 Introduction

Emulsion polymers serve as additives for a range of applications, including adhesives, binders in coatings and paints, and modifiers in cement mixtures. In the last application, these polymers are often preferred in dry form due to various benefits, such as lower packaging, transportation and storage costs, extended shelf life, and the convenience of dry-blending to create single-bag mixtures. However, a crucial requirement is that the dry polymer must have the ability to redisperse upon contact with water, yielding a new emulsion providing good polymer distribution within the application formulation.^1–5^ A successful example is that of the polymer powders produced by spray-drying emulsion polymers that have been utilized as additives for mortars, enhancing both the fresh and set mortar’s performance. These dry polymer powders can be easily blended with a dry mortar, offering a convenient single-bag formulation. On-site, the contents of the bag can be mixed with water, allowing for the restabilization of latex particles within the wet mortar. The improved performance achieved through the use of polymeric binders in construction applications is attributed to the cohesive bonding of polymers with inorganic materials (cement, gypsum, lime, sand, etc.) in the formulation, resulting in the formation of a flexible and hydrophobic network.^4–6^ Dispersions of soft polymers of low glass transition temperatures (*T*_g_ < 20 °C) such as styrene acrylics, styrene butadiene, and copolymers of vinyl acetate (VAc) with ethylene or VeoVa10 are used industrially. Poly­(vinyl alcohol) (PVOH) is often included in the latex formulation as a colloidal stabilizer. Upon drying, the PVOH forms a hard shell covering the soft particles and hence preventing their interdiffusion during drying and storage.^4,5,7–9^ Additionally, inorganic fillers such as kaolin are employed as anticaking agents to prevent the fusion of sticky aggregates, thereby ensuring the production of a free-flowing powder product.^4,8,10,11^

The current study aims at expanding the concept of dry latex polymers by integrating stimuli-responsive stabilization methods capable, on one part, of being deactivated, causing the coagulation of the polymer particles, and on the other, of being activated to redisperse the coagulated particles. This technology relies on two crucial characteristics: the development of a switchable colloidal stabilizer to achieve the coagulation/redispersion cycle and the particle surface protection using a hard shell to prevent particle coalescence during coagulation, drying, and storage.

Switchable stabilization can be achieved through the incorporation of surface-responsive moieties, such as tertiary amines, amidines, and carboxyl groups. In this regard, it is worth pointing out that PVOH that is the current technology for the production of dry polymers is not responsive. The inclusion of tertiary amines and amidines in the colloidal stabilization system renders the particle surface-responsive to changes in pH and atmosphere (N_2_/CO_2_). This approach has been studied in the context of synthesizing redispersible latexes by various research groups.^12–22^ The amine groups are protonated and positively charged below the p*K*_a_ value, providing electrostatic stabilization. At pH > p*K*_a_, the amines become neutral, leading to latex coagulation. Decreasing the pH through the addition of acid or by acidifying the aqueous medium by CO_2_ bubbling can restabilize the particles through the protonation of amine groups, inducing redispersion. Cunningham and co-workers have utilized responsive amidine-based surfactants and azo-based initiators in the emulsion polymerization of polystyrene (PS) and poly­(methyl methacrylate) (PMMA) latexes.^13,15,17^ Redispersion of the coagulated particles was achieved by using sonication and CO_2_ bubbling. The high *T*_g_ of the polymers used limited the degree of particle deformation and interdiffusion, facilitating the redispersion. Zhang et al.^14^ and Darabi et al.^21^ applied this idea to softer latexes (*T*_g_ ≈ 20–25 °C). Redispersibility was achieved with freshly formed wet aggregates (*i.e*., without water removal and drying). Drying the aggregates of soft polymers resulted in particle fusion, making them unable to redisperse.^18^ In addition, these systems are not practical in applications of high pH such as construction due to their p*K*_a_ range (8–11), which leads to coagulation. Consequently, a system with maximum stability at the applied pH is required.

Carboxyl groups^23,24^ offer maximum stability at the basic pH of the application in construction. Wang et al. synthesized a three-layer polymer particle consisting of PBA (poly­(butyl acrylate)) as the soft core, PS (polystyrene) as the hard shell, and PMAA (poly­(methacrylic acid)) as surface-responsive moieties, resulting in a redispersible latex (without drying).^25^ The method used was reversible addition–fragmentation chain transfer (RAFT), which can lead to high costs. In a more straightforward method, carboxyl surface functionalization was achieved by simply incorporating acidic functional comonomers (*e.g*., methacrylic acid, itaconic acid, etc.) into the formulations used in spray-dried systems to enhance the stability of the reconstituted latex from the powder. These formulations also included the protective colloid (PVOH) and anticaking agent (calcium carbonate or silica sol).^26,27^ Although these formulations were not specifically studied for coagulation and restabilization, they offer a potential route for incorporating carboxyl moieties into the synthesis of switchable systems. Naderi et al. synthesized pH-responsive latexes of 30 and 40% solid contents and low *T*_g_ (17 °C) via surfactant-free semicontinuous emulsion polymerization.^28^ In the process, the reactor was initially charged with an aqueous solution of initiator (potassium persulfate), and the monomer mixture (vinyl acetate, vinyl neodecanoate, and methacrylic acid, VAc/VeoVa10/MAA = 55/45/1–2.5 wt/wt/wt) was fed semicontinuously. This led to the *in situ* formation of surface acid groups, providing the particles with pH responsiveness and relative surface hardness. Reduction of the pH led to coagulation. The polymer was isolated by filtration and then dried. The dry product could be effectively restabilized in water when a minimum amount of MAA was used (2 wbm%). Despite the straightforward *in situ* surface functionalization method used, it was found that only between 32 and 47% of the acid groups were incorporated on the surface of the particles. The rest remained as water-soluble species in the aqueous phase or were buried inside the particles.

A drawback of Naderi′s approach^28^ is that the resistance to caking during storage is limited for the low *T*_g_ latexes needed for the application in construction. A way to overcome this limitation is to use soft core–hard shell particles.^26,27^ However, the hard shell might hinder the film-forming ability, which is crucial for application as binder such as construction.^29^ Actually, similar soft core–hard shell systems are prone to cracking.^30–33^

Alkali-soluble resins (ASRs) appear to be promising candidates as they simultaneously offer stability, surface protection, and responsiveness. These resins are random copolymers composed of hydrophobic hard monomers (such as styrene, methyl methacrylate, and α-methylstyrene) and hydrophilic comonomers (such as methacrylic acid and acrylic acid). They are often used as stabilizers for polymerization in dispersed media.^34–39^ Their high *T*_g_ provides the surface of the particles with a protective layer.^40,41^ Additionally, the acid moieties in the chain render the ASRs pH-responsive, making them potential switchable protective colloids for the control of coagulation and restabilization. A useful aspect of ASRs is that in the dry state, they are rigid, forming a hard shell around the particles, while in the presence of water, they become hydroplasticized, significantly reducing their effective *T*_g_, which improves the film-forming ability of the latex particles.^41,42^ This behavior is well-aligned with applications wherein the rigid ASR shell can protect the particles from deformation in the dry form without hindering film formation. In addition, using ASRs in the form of particles as seeds for the emulsion polymerization of the main monomer system might lead to higher incorporation of surface acid groups compared to using a functional monomer through an *in situ* method.^28^

This study aims to address the challenges and limitations of switchable latex systems such as ensuring maximum stability under application conditions, providing effective surface protection without hindering film formation, and improving surface incorporation of responsive moieties. By exploring the potential of alkali-soluble resins (ASRs) as pH-responsive protective colloids, this work introduces a versatile solution for achieving reversible particle coagulation and restabilization. Utilizing a seeded semicontinuous emulsion polymerization process, latexes based on 55 wt % VAc and 45 wt % VeoVa10 were synthesized (target *T*_g_ = 14 °C), with ASRs serving as both seed particles and stabilizers. The resulting latexes were coagulated at low pH for polymer isolation, followed by filtering, drying, grinding, and reinforcement with fillers to obtain a fine powder. This research explores how varying ASR compositions impact latex synthesis, particle stability, surface acid group incorporation, and restabilization behavior (particle size recovery and sediment content) under different temperatures and pH conditions. The findings provide insights into the development of switchable polymeric latexes for applications under high pH conditions such as construction.

## 2 Experimental Section

To synthesize pH-responsive ASR-based latexes, a seeded process is proposed, starting with the synthesis of ASR seeds through semicontinuous emulsion polymerization. ASR seed latexes with varying acid monomer (MAA) contents of 15, 25, and 35% were produced and named ASR15, ASR25, and ASR35, respectively. Subsequently, the seed latexes were charged by increasing the pH, thereby deprotonating the acid moieties. The particles were then grown by polymerizing the main monomer system (55 wt % VAc–45 wt % VeoVa10) inside the seed (Figure 1). In this process, 10 wbm% of different seeds were employed, leading to the synthesis of latexes with distinct MAA contents, denoted as 10ASR15, 10ASR25, and 10ASR35.

**Figure 1:**
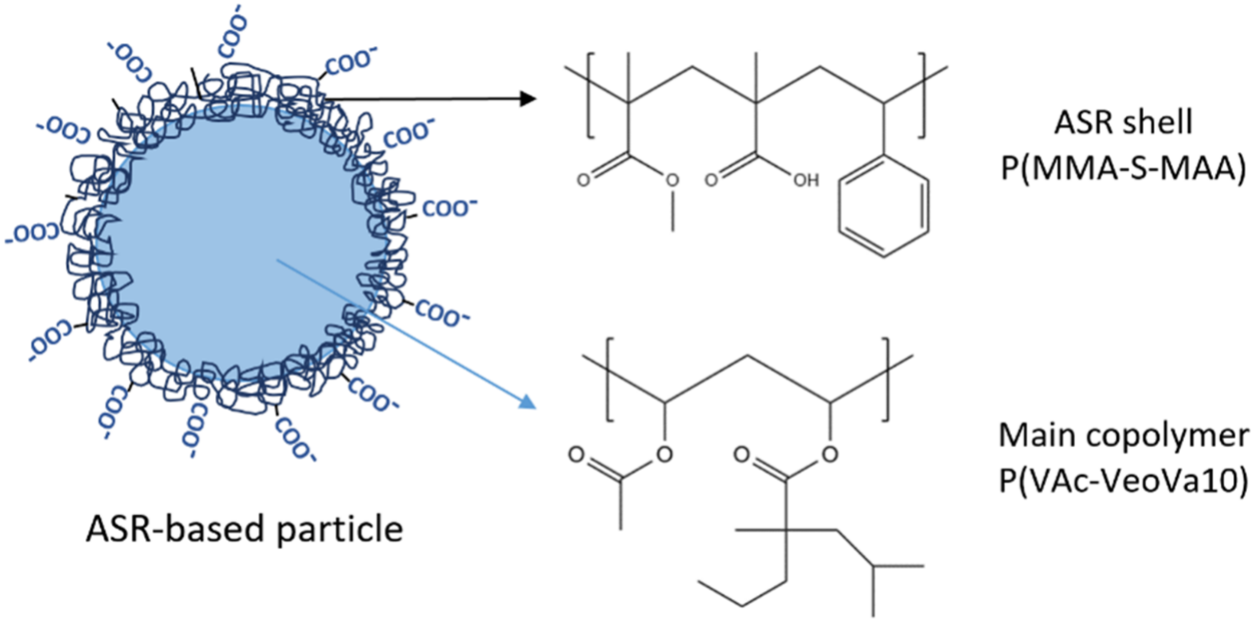
Schematic of the ASR-based emulsion particle and its composition.

### 2.1 Materials

The monomers used, including vinyl acetate (VAc, Quimidroga), vinyl neodecanoate (VeoVa10, Hexion), methyl methacrylate (MMA, Quimidroga), styrene (S, Quimidroga), and methacrylic acid (MAA, Sigma-Aldrich), were employed without further purification. Potassium persulfate (KPS, purity ≥ 99%, Sigma-Aldrich) served as the thermal initiator. Sodium dodecyl sulfate (SDS, Sigma-Aldrich) was utilized as the surfactant for the emulsion polymerization of ASRs. 2-Ethylhexyl thioglycolate (EHTG, Sigma-Aldrich) was used as a chain transfer agent (CTA) to target the desired molecular weight of the ASRs. Hydrochloric acid (HCl, Sigma-Aldrich) and sodium hydroxide (NaOH, Scharlab) were employed to adjust the pH. Deionized water was used as the reaction medium in the emulsion polymerizations. A reference polymer powder based on VAc and ethylene, having a *T*_g_ of 16 °C (ref-VAE), was kindly supplied by Wacker Chemie AG. Tetrahydrofuran (THF, GPC grade, Scharlab) served as the solvent in gel permeation chromatography (GPC) and gas chromatography (GC) studies. Acetic acid and 1-pentanol from Sigma-Aldrich were used in GC calibration and measurements. Kaolin powder (Sigma-Aldrich) was used as an anticaking agent.

### 2.2 Synthesis of ASR Seeds

ASRs of different compositions, featuring high glass transition temperatures (*T*_gs_) and varying acid concentrations (Table 1), were synthesized through semicontinuous emulsion polymerization using the formulations in Table 2. In the synthesis, the glass reactor (1 L) was charged initially with the initial charge of water and SDS and purged with N_2_ for 10 min under stirring at 250 rpm, using a stainless steel anchor-type stirrer. The temperature was then raised to 80 °C, and an aqueous solution of the initiator (KPS) was added as a shot to the initial charge. After 5 min, the preemulsion including water, SDS, monomers, and EHTG was fed over 90 min. The preemulsion was stirred using a magnetic stirrer during feeding. The target final solids content at full conversion was 22 wt %. After feeding, the reaction continued for 120 min to reduce the residual monomer. Finally, the reaction was cooled to room temperature.

**Table 1:** ASR Compositions and Their Theoretical *T*_g_ and Acid Values (AV)

	composition (wt %)				
ASR name	MMA	*S*	MMA	*T*_g_ (°C)	AV (mg KOH/g sample)
ASR15	80	5	15	119.5	97.9
ASR25	70	5	25	129.8	163.2
ASR35	60	5	35	140.6	228.4

**Table 2:** Formulations Used to Synthesize ASRs of Different MAA Contents

		latex name		
	material^a^	ASR15	ASR25	ASR35
initial charge	water	514	514	514
	SDS	0.2	0.2	0.2
initiator shot	water	50	50	50
	KPS	1	1	1
preemulsion	water	150	150	150
	MMA	160	140	120
	S	10	10	10
	MAA	30	50	70
	EHTG	7.3	7.3	7.3
	SDS	0.4	0.4	0.4

a All values are in grams.

### 2.3 Synthesis of ASR-Based Latexes

The ASR latexes were utilized as seeds to synthesize low *T*_g_ latexes (VAc/Veova10, 55/45 wt/wt). The formulations are presented in Table 3. Initially, the glass reactor (1 L) was charged with water and ASR latex. An aqueous solution of NaOH (5 wt %) was added dropwise to achieve a pH of around 6.5, above the p*K*_a_ of carboxylic groups (pH ≈ 4.5 ^43,44^ ) in order to deprotonate the MAA groups. Subsequently, the temperature was raised to 70 °C with stirring at 250 rpm under a nitrogen flux. The initiator was added as a shot, and after 5 min, the monomer mixture was fed over 3 h. A base solution was fed in a separate stream for 3 h to control the pH during the reaction. For 10ASR25 and 10ASR35, the reaction pH was around 5.5–6.5, while for ASR15, a higher amount of NaOH was fed to maintain the pH around 6.5–7 throughout the reaction, since coagulation was observed at lower pH. This difference can be attributed to the higher acid content in ASR25 and ASR35 compared to ASR15, as well as the greater degree of chain extension (steric stabilization), which contributed to superior stability at similar pH levels. After feeding, the reaction continued for 120 min, followed by cooling to room temperature. The final solids content at full conversion was 40 wt %.

**Table 3:** Formulations Used to Synthesize ASR-Based Latexes of Different MAA Contents

		latex name		
	materials^a^	10ASR15	10ASR25	10ASR35
initial charge	water	260.2	318	318
	ASR15 latex	136.4		
	ASR25 latex		136.4	
	ASR35 latex			136.4
	NaOH 5 wt % solution	12	8	8
initiator shot	water	50	50	50
	KPS	1.5	1.5	1.5
monomer mixture	VAc	165	165	165
	VeoVa10	135	135	135
base solution	NaOH	4	0.9	0.9
	water	76	17.1	17.1
starting pH		6.71	6.54	6.41

a All values are in grams.

It is worth pointing out that the reason for using the ASRs in seed form was to achieve better stability. Utilizing ASRs fully dissolved in the initial charge (*e.g*., pH > 7.5 for ASR35) resulted in a final latex of micron-sized particles and coagulation (15 wt % sediment at the end of polymerization), possibly due to poor desorption of ASR as a polymeric surfactant to stabilize the newly formed particles.^45^

### 2.4 Polymer Isolation

The coagulation process is illustrated in Figure 2. This procedure involved the gradual addition of a 2 wt % aqueous solution of hydrochloric acid (HCl) using a syringe pump at a rate of 0.4 mL/min to 100 g of latex while mechanical stirring utilizing a 6-bladed Rushton turbine impeller at 300 rpm. Throughout the process, the pH was monitored by using a pH probe. Samples were withdrawn during destabilization, and their viscosity was measured by using a parallel plate rheometer. Acid addition was halted upon the detection of a significant increase in viscosity due to rapid coagulation, resulting in the formation of a viscous slurry. The HCl fed neutralized the excess of NaOH used to control the reaction pH, acetic acid from the hydrolysis of vinyl acetate hydrolysis during the reaction, and COOH groups. The amount consumed to protonate the COOH groups was

1 X=HCladded−NaOH+aceticacid(mol)

**Figure 2:**
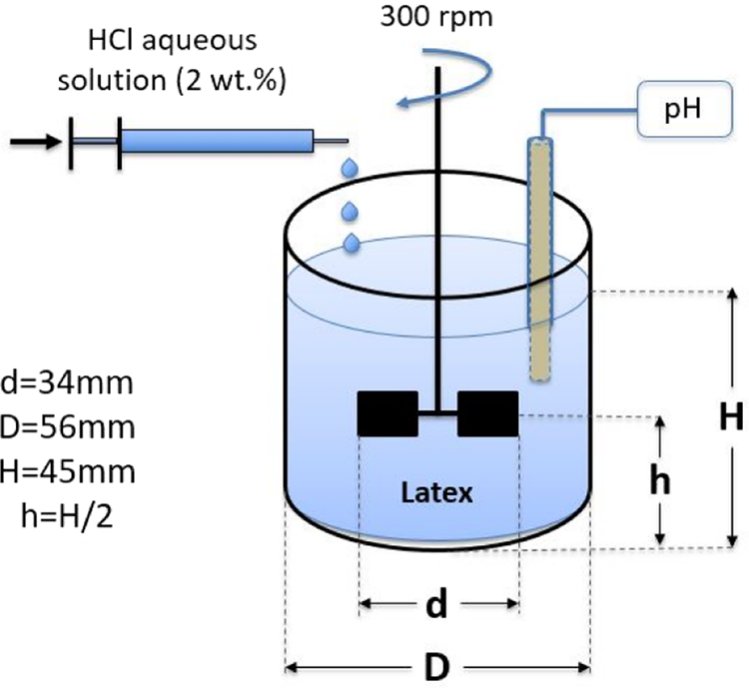
Schematic of the coagulation process.

50 g of the resulting slurry was filtered for 30 min using a suction flask connected to a vacuum pump with a vacuum ranging from 200 to 700 mbar during the filtration time. Approximately 70% of the water was successfully filtered, leaving the sample with a reduced water content for subsequent evaporation. The resulting wet slab (Figure 3a) was collected and crumbled using a spatula to achieve millimeter-sized flakes and aggregates (Figure 3b). Subsequently, the aggregates were dried for around 8–10 h on aluminum foil at 23 ± 2 °C and 50 ± 5% relative humidity until reaching a water content of about 1 wt %. During drying, the sample was ground every 2 h using a kitchen coffee grinder to obtain submillimeter-sized dry polymer aggregates. In each grinding step, the sample was ground in 3 cycles of 5 s of grinding and 5 s of rest (Figure 3c). The sample was stored for 1 day prior to being assessed for restabilization.

**Figure 3:**
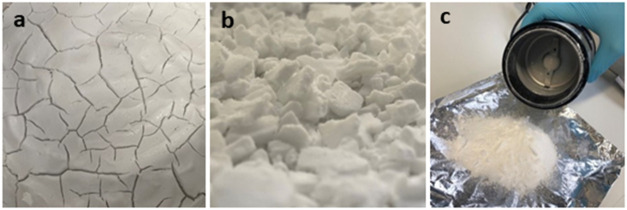
(a) Wet aggregate remaining after filtration, (b) millimeter-sized crumbled aggregates, and (c) fine dry polymer aggregates after last grinding.

### 2.5 Dry Polymer Aggregates Reinforcement

To enhance the temperature and caking resistance of the polymer aggregates, two different strategies were employed to incorporate kaolin as a reinforcing agent in the formulation, namely, wet and dry mixing. In the wet mixing approach, 20 g of kaolin was dispersed in 80 g of water in a 100 mL glass beaker using an Ultraturrax (T 25 digital ULTRA-TURRAX homogenizer from IKA equipped with the S 25 N–25 F dispersing tool) at 5000 rpm for 10 min. Then, 16 g of the kaolin dispersion was then added to 100 g of the latex (to achieve 8 wt % of kaolin based on the polymer) under magnetic stirring at 600 rpm for 30 min. The resulting dispersion was subsequently coagulated, dried, and ground to obtain a dry powder reinforced with kaolin. In the dry mixing strategy, 8 wt % of kaolin powder and the dry polymer were blended by adding kaolin in the last grinding step after drying. The strategies are summarized in Table 4.

**Table 4:** Strategies Used to Incorporate Kaolin in Reinforced Formulations

strategy	filler phase	polymer phase	mixing method
wet mixed	kaolin dispersion (20 wt %) by ultraturrax	latex	magnetic stirring (600 rpm)
dry mixed	kaolin powder as received	dry aggregates	grinding by coffee grinder

### 2.6 Restabilization

The restabilization involved mixing 20 g of the dry polymer aggregates with 80 g of water, followed by dropwise addition of a 5 wt % aqueous solution of NaOH to achieve a redispersion pH of approximately 9, aiming to restore the charged moieties. The mixing was performed under high shear conditions (stirring rate of 5000 rpm) for 30 min using the Ultraturrax mentioned in Section 2.5. Samples were collected at 10 min intervals, and the recovered particle size of the stable portion, along with the sedimentation percentage after a 3 h rest, were measured. The sediment is considered as the fraction that could not be restabilized effectively.

### 2.7 Characterizations

The ASRs, ASR-based latexes, isolated polymer aggregates, and restabilized latexes underwent different characterizations. Detailed descriptions of the methods used are provided in Section SI.1.

Gravimetric analysis was used to determine the solids content and instantaneous monomer conversion (*X*_inst_) as follows

2 $$solids\;content\left(t\right)=\frac{weight\;of\;the\;solids\left(t\right)}{weight\;of\;the\;latex\;sample\left(t\right)}$$

3 $$X_{inst}\left(t\right)=\frac{solids\;content\left(t\right)-W_{s}\left(t\right)}{W_{m}\left(t\right)}$$

where *W*_s_(*t*) is the fraction of initiator, ASR and NaOH in the reactor and *W*_m_(*t*) is the fraction of fed monomer at the sampling time *t*.

Dynamic light scattering (DLS) was used to determine the particle size distribution (PSD). Gas chromatography (GC) was employed for examining the degree of hydrolysis (DH) by measuring acetic acid resulting from vinyl acetate hydrolysis.^28^ Water-soluble species content (WSC) was measured by drying the supernatant obtained from centrifugation. Additionally, the molecular weight of the ASRs was measured by using size exclusion chromatography (SEC). A complete description of the SEC setup and conditions is provided in the SI.

The viscosity of the samples withdrawn during the coagulation process was measured using a parallel plate rheometer (AR1500ex). Sedimentation of the restabilized latexes was quantified by weighing the amount of dried sediment after 3 h from restabilization. The glass transition temperature (*T*_g_) of the ASR-based polymer was determined by using differential scanning calorimetry (DSC). Laser diffraction (Malvern Mastersizer) was employed to determine the sizes of the powder aggregates.

To assess the impact of temperature on restabilization, the dry polymers were subjected to different temperatures (30, 40, and 50 °C) in an oven for 1 day before restabilization. To evaluate the effect of the pressure, a caking test was devised to simulate the pressure that dry polymer aggregates may experience during storage. In this test, 20 g of dry polymer was placed in a glass beaker (30 mL, diameter = 2.9 cm), and a pressure of 14.9 kPa was applied by placing a 1 kg steel cylinder on top. The sample was then positioned in an oven at 50 °C for 1 day, and the caking behavior of the dry polymer aggregates was assessed through visual observation. The dry samples subjected to different conditions were restabilized in water. After 3 h, the sedimentation and the recovered particle size of the stable portion were measured.

To evaluate the core–shell morphology of the ASR-stabilized latexes, differential scanning calorimetry (DSC) and TEM analysis were carried out.

## 3 Results and Discussion

### 3.1 Latex Synthesis

Table 5 shows the characteristics of the ASR latexes (used as seeds to produce the final latexes). High conversions (approximately 99%) were achieved in all cases. Particle sizes ranging from 170 to 200 nm were obtained. The weight-average molecular weights of the ASRs ranged between 12,500 and 14,300 Da. The ASR latexes were pH-responsive, dissolving in water at a high pH (>8) and precipitating at a low pH (<3).

**Table 5:** Characteristics of ASR Seed Latexes

	composition (wt %)					
latex	MMA	*S*	MAA	dp (nm)	conversion (%)	MW (Da)
ASR15	80	5	15	190	99.3	14,300
ASR25	70	5	25	200	99.2	12,500
ASR35	60	5	35	170	98.7	12,500

Figure 4 illustrates the evolution of conversion, particle size, and number of particles during the reactions stabilized by different ASRs. The polymerizations were carried out under starved conditions, achieving similar instantaneous conversions for all of the reactions. While the number of particles of 10ASR35 increased during the reaction due to the formation of new particles, there was a decrease in the number of particles of 10ASR15 and 10ASR25 as a result of flocculation. This difference can be attributed to the higher stabilization capability and water solubility of ASR35 due to its higher acid content compared to those of ASR15 and ASR25. This enhances the ability to stabilize newly formed particles, resulting in a smaller final average particle size.

**Figure 4:**
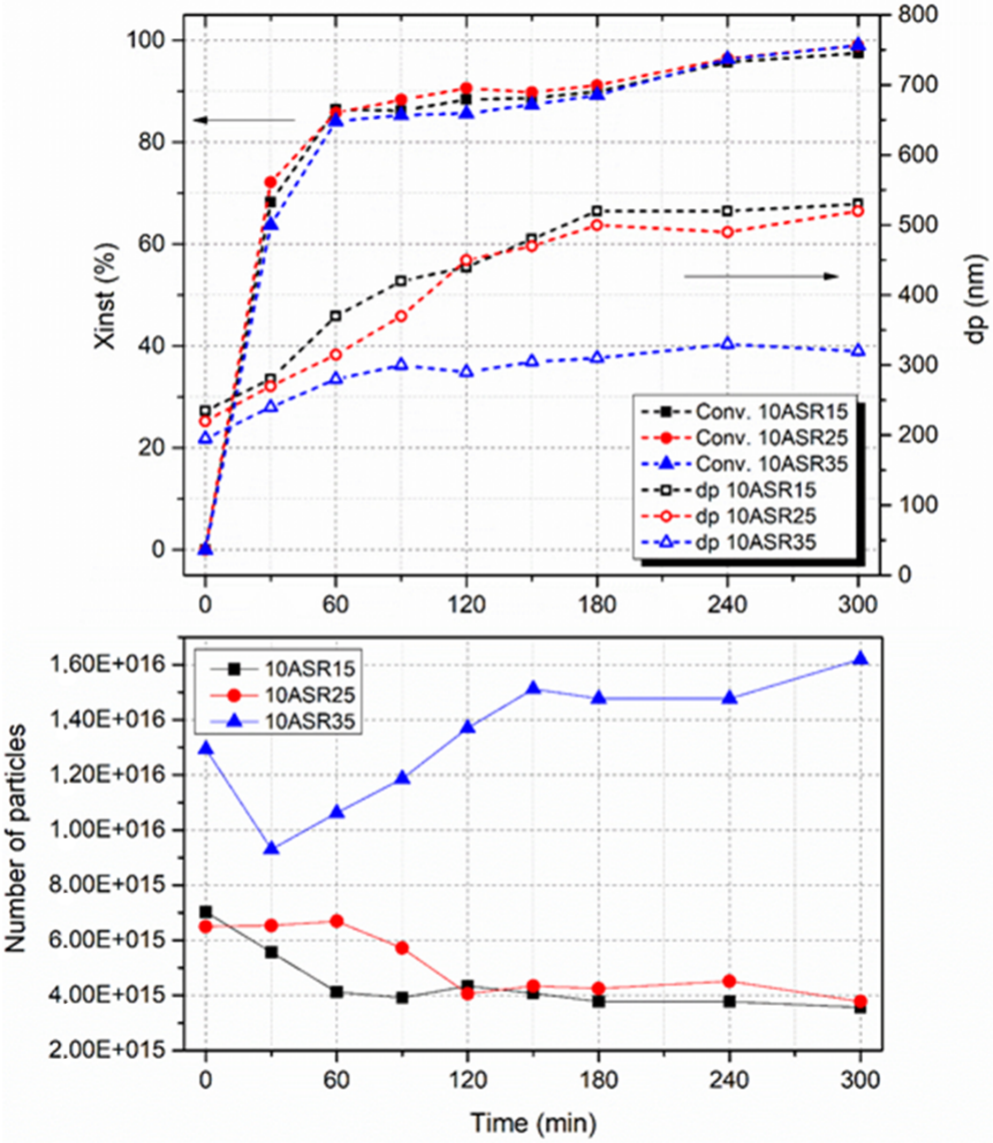
Evolution of instantaneous conversion, particle size, and number of particles during emulsion polymerization of ASR-based latexes of varying MAA contents.

Table 6 details the characteristics of the final ASR-stabilized latexes (55VAc/45VeoVa wt/wt %, *T*_g_ = 14 °C). High conversions and relatively large particle sizes (controlled through the seed size) were achieved in all cases. The rationale behind using large seeds (as those of Table 5) was to obtain large final particles in order to decrease the total particle surface area and increase the thickness of the ASR layer. Thus, using small 85 nm ASR seeds of 15 wt % MAA (smaller than those used in Table 5) resulted in a 190 nm latex size, which exhibited poor restabilization (41 wt % sedimentation) due to the thin surface ASR layer that did not provide adequate surface protection.

**Table 6:** Characteristics of ASR-Based Latexes with Varying MAA Contents

latex	dp (nm)	solids content (wt %)	conversion (%)	final pH	DH^a^ (%)	WSC^b^ (wbm %)
10ASR15	530	39	97.1	6.81	3.22	1.98
10ASR25	520	39.6	98.6	5.86	0.95	1.17
10ASR35	320	39.7	98.9	5.66	0.93	1.36

a Degree of hydrolysis based on total VAc amount in the system.

b Water-soluble species content, excluding NaOH amount used. 0.5 wbm % KPS was used as an initiator, which contributes in water-soluble species content.

The higher pH needed for the synthesis of the latex stabilized by ASR15 led to a higher degree of hydrolysis compared with those of 10ASR25 and 10ASR35 (<1%). In addition, all of the latexes were film-forming at ambient conditions (not hindered by the ASR shell) having a minimum film formation temperature (MFFT) of about 10 °C, which is suitable as a binder.

The surface incorporation of ASRs, and consequently acid groups, was analyzed by measuring the water-soluble species separated through ultracentrifugation. This analysis accounted for both soluble ASR chains in water and the contributions from NaOH and the remaining KPS used in the reaction. The reported values exclude the NaOH content. More than 80% of ASR chains (*i.e*., acid groups) were incorporated into the particles, confirming a superior surface incorporation of acid groups compared to the previous work,^28^ where using a functional acidic monomer in a surfactant-free emulsion polymerization resulted in less than 50% surface incorporation.

10ASR15, performed at pH 6.5–7, exhibited a higher amount of water-soluble species (*i.e*., higher ASR content in water), reflecting increased solubility of ASR chains in water at a higher pH. Under comparable reaction pH values, 10ASR35 had a slightly higher content of water-soluble species than 10ASR25, which correlates with the higher MAA content in the ASR and increased solubility.

Further assessment of the incorporation of the ASR in the shell; namely, the formation of core–shell morphology particles was sought by using DSC and TEM (see Figures S1 and S2 and Section SI.2). DSC was not conclusive because a single *T*_g_ corresponding to the VAc/Veova copolymer is observed likely due to the low amount of ASR (10 wt %) employed in the formulation. On the contrary, TEM images show dark lines surrounding deformed particles, indicating a core–shell morphology of the particles.

### 3.2 Polymer Isolation

Coagulation of the latexes was achieved by addition of a 2 wt % aqueous HCl solution while monitoring the pH evolution using a pH probe. The viscosity of the samples withdrawn during coagulation was checked by using a parallel plate rheometer. Figure 5 illustrates the viscosity and pH changes during the destabilization of the 10ASR15 latex. The pH gradually decreased during acid addition, and around pH 4, the decline intensified, followed by coagulation at pH = 3, resulting in a jump in viscosity due to the rapid formation of large aggregates. All of the latexes were successfully destabilized at pH = 3 by the protonation of the MAA groups. The slurries obtained from coagulation were filtered, dried, and ground to obtain ASR-based dry polymers for subsequent restabilization studies.

**Figure 5:**
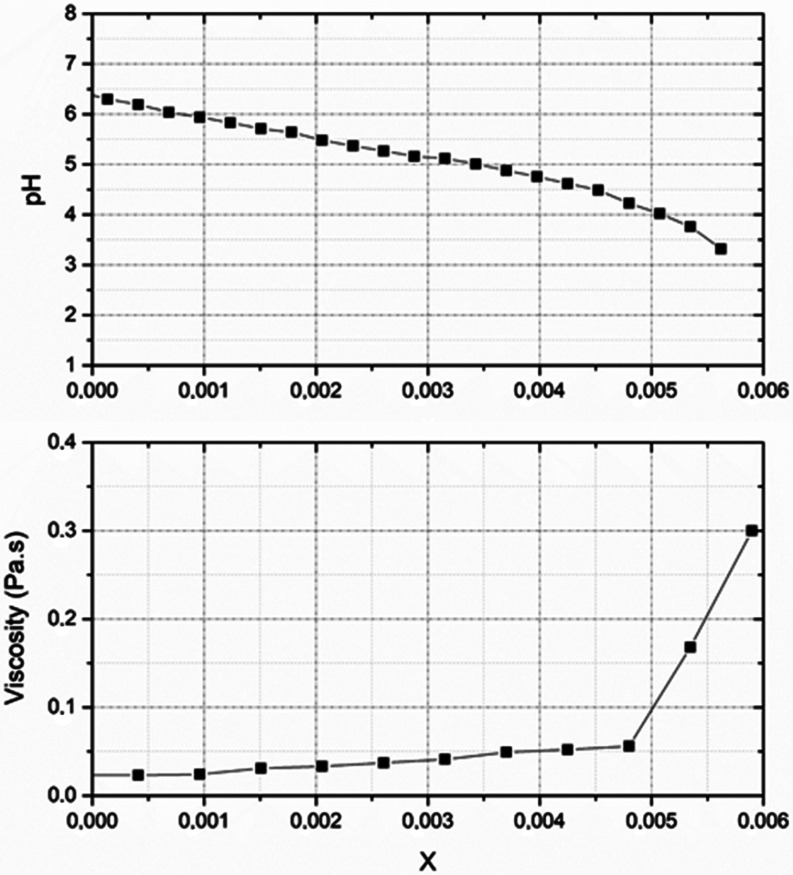
pH and viscosity change during destabilization of the ASR-based latexes of varying MAA contents. *X* is the mole of added HCL consumed to protonate the MAA units.

### 3.3 Restabilization

All dry polymer samples were successfully restabilized in water under 30 min of high shear conditions provided by the Ultraturrax. Less than 10 wt % of sedimentation and recovered particle sizes ranging from 460 to 550 nm (Table 7), which were lower than the reference sample, were obtained. Restabilization improved by increasing the Ultraturrax time and with a higher acid content in the ASR, where a higher number of carboxyl groups led to a more efficient restabilization. Samples 10ASR15 and 10ASR25 exhibited recovered particle sizes similar to their original latex, while 10ASR35 showed a 44% increase in the recovered particle size compared with its original latex, despite having the highest acid content. This may be due to the smaller initial particle size of 10ASR35, resulting in a thinner surface ASR layer. Additionally, smaller particle sizes lead to a higher number of particles within the aggregates, creating more contact points and increased cohesion between particles,^32^ which can complicate the release of the primary particles. Furthermore, a low extent of VAc hydrolysis occurred during restabilization, ranging between 0.35 and 0.52% based on total VAc groups. This can be attributed to the shielding effect of the VeoVa10 units.^46^

**Table 7:** Restabilization Features of ASR-Based Dry Polymers of Varying MAA Contents

		mixing time by ultraturrax^a^ (min)		
sample original dp	features	10	20	30
10ASR15 530 nm	R-dp (nm)	560	540	550
	3 h-sed^b^ (%)	19.3	9.2	7.8
	DH (%)			0.35
10ASR25 520 nm	R-dp (nm)	570	540	540
	3 h-sed (%)	6.8	4.1	3.4
	DH (%)			0.47
10ASR35 320 nm	R-dp (nm)	480	460	460
	3 h-sed (%)	6.1	3.7	2.8
	DH (%)			0.52
ref-VAE	R-dp (nm)			1000
	3 h-sed (%)			16.5
	DH (%)			0.23^b^

a Stirring at 5000 rpm.

b Based on total dry polymer amount.

The restabilization of the isolated polymer (55 wt % VAc–45 wt % VeoVa10) obtained in this work using ASR as the stabilizer was compared with that of the latexes obtained in ref 28. In ref 28, carboxylated latexes with varying percentages of MAA (1, 1.5, 2, and 2.5 wbm%) were synthesized via surfactant-free semicontinuous emulsion polymerization of VAc and VeoVa10. The resulting particle sizes ranged from 300 to 500 nm, with smaller particle sizes observed as the MAA percentage increased.^28^ Figure 6 compares the restabilization behavior and surface concentration of MAA groups, measured by titration for the carboxylated latexes and calculated theoretically based on the water-soluble species content for the ASR-based latexes. It must be pointed out that the contribution of KPS in water-soluble species was neglected in the calculations for ASR-based latexes, meaning that the MAA surface concentration values are underestimated. The ASR-based latexes exhibited a higher surface concentration of switchable moieties, resulting in improved restabilization (lower sedimentation and smaller recovered particle sizes) compared with the carboxylated latexes. This might be the result of two contributions. First, the large particle size and better surface incorporation of the ASRs led to better restabilization. Additionally, ASRs provide a rigid protective shell on the particle surfaces, which hinders particle sintering during latex coagulation and drying and facilitates restabilization from the dry form.

**Figure 6:**
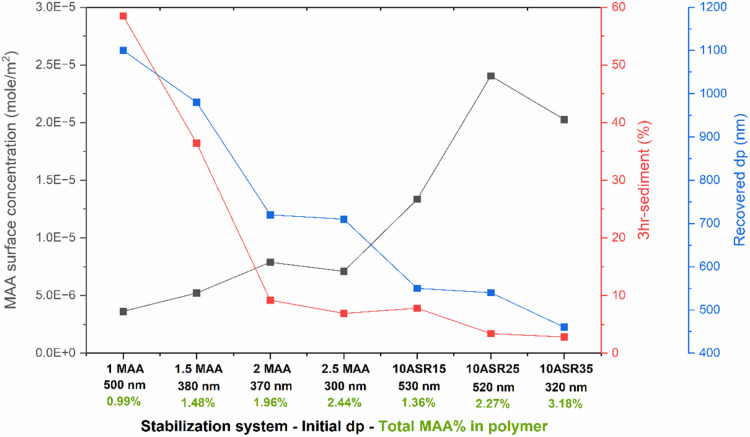
Surface concentration of MAA groups and restabilization behavior comparison of carboxylated and ASR-based latexes.

#### 3.3.1 Effect of Temperature and Pressure on Restabilization

The restabilization behavior of the dry polymer (10ASR35) exposed to elevated temperature and pressure—conditions that a dry product may experience during storage—was studied. Dry pristine 10ASR35, subjected to various temperatures for 1 day, was restabilized in water, and the characteristics of the recovered dispersions are presented in Figure 7 (black points). Restabilization declined significantly (higher sedimentation and larger recovered particle sizes) by increasing the temperature, likely due to increased particle deformation and a higher probability of sintering. Dry addition of 8 wt % kaolin (commonly used industrially) to the dry polymer powder in the last grinding step significantly improved the temperature resistance (Figure 7, red points), and there was only a slight increase of 2–4% in the sedimentation by increasing the temperature. It is important to note that the sediment included micron-sized fast-sedimenting kaolin particles, leading to an overestimation of the values presented for the reinforced samples. Consequently, at low temperatures, the apparent effect was that the pristine sample performed better than the reinforced samples. An example of the higher contribution of the inorganic filler in the sediment is shown in Section SI.3.

**Figure 7:**
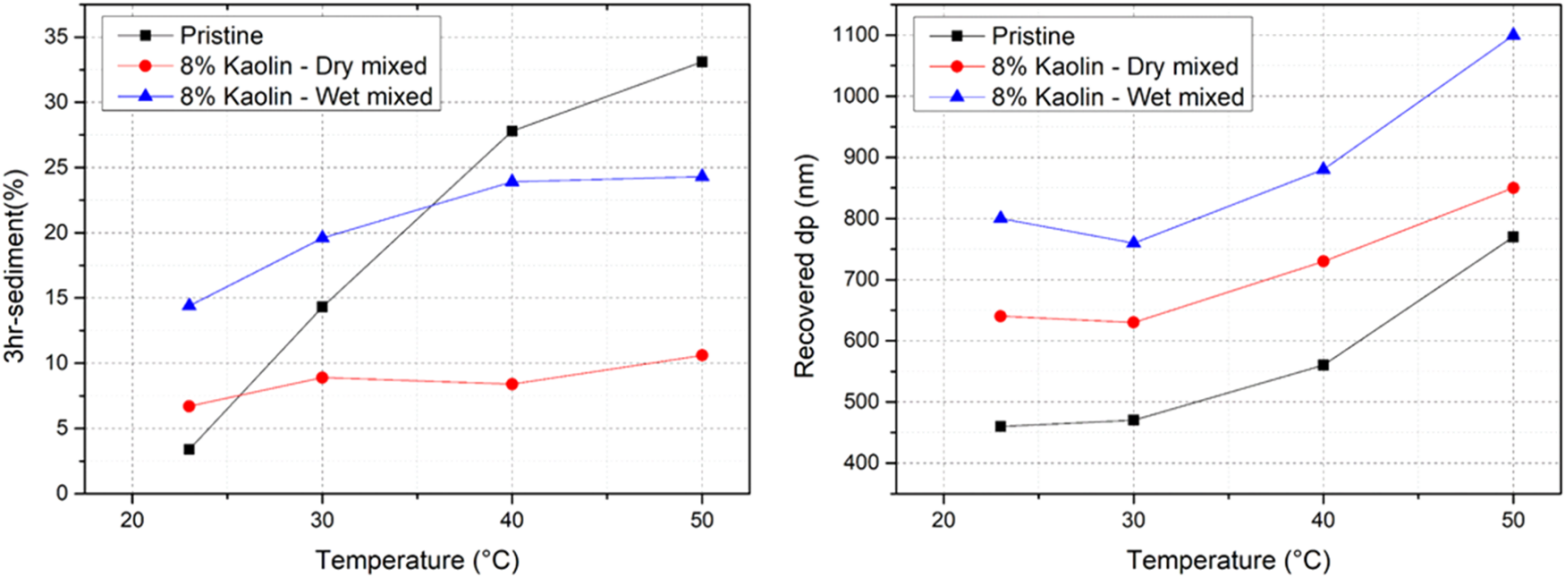
Effect of temperature on restabilization of the pristine and kaolin-reinforced ASR-based samples (10ASR35—initial dp: 320 nm).

In addition, kaolin significantly enhanced the free-flowing behavior of the dry product, as shown in Figure 8. Exposing the pristine sample to 50 °C for 1 day resulted in a blocky material due to the increased stickiness of the polymer aggregates and their sintering upon contact. In contrast, the kaolin-reinforced sample maintained its free-flowing behavior (moving downward in a vertical position, Figure 8) because the rigid kaolin particles acted as an anticaking agent, covering the polymer aggregates and preventing their surfaces from coming into contact.

**Figure 8:**
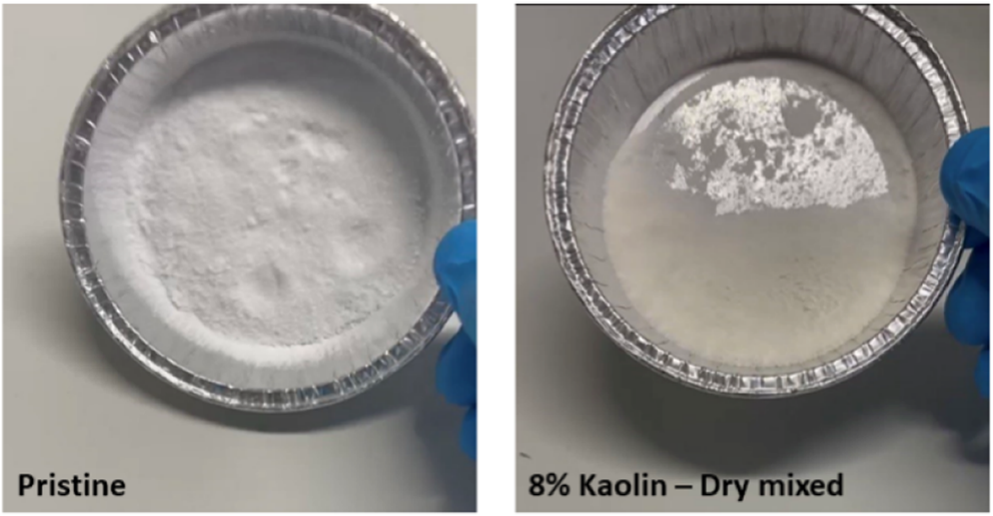
Appearance of the pristine (left) and kaolin-reinforced (right) ASR-based polymer (10ASR35) after exposure to 50 °C for 1 day.

Polymer reinforcement by wet mixing kaolin with the latex prior to coagulation (Figure 7, blue points) was less effective in enhancing the temperature resistance compared to dry mixing. This difference can be attributed to the pH responsiveness of kaolinite, the primary component of kaolin.^47–49^ When kaolin is mixed with the ASR-based latex and then subjected to coagulation, the reduction in pH decreases the charge density and stability of the kaolin particles, leading to their agglomeration and a decline in the total surface area. This limits the efficiency of kaolin as a reinforcing filler for covering polymer aggregates. To support this, ζ-potential and stability measurements of kaolin dispersions at different pH levels were conducted, with results presented in Section SI.4.

Kaolin-reinforced samples, both dry and wet mixed, exhibited larger recovered particle sizes compared to the pristine polymer, likely due to the presence of large, stable, negatively charged kaolin particles in the basic pH dispersion.

To investigate the influence of pressure on restabilization, kaolin-reinforced dry polymers with low and high acid contents (10ASR15 and 10ASR35, respectively) were exposed to different conditions, including ambient temperature, 50, and 50 °C under a 14.9 kPa pressure for 1 day. After the caking test, the polymer aggregates could be broken easily using a spatula and preserved their free-flowing behavior. Following exposure, restabilization was performed using the Ultraturrax, and the results are summarized in Table 8. Increasing temperature and applying pressure promote particle deformation and sintering, significantly raising the amount of sediment. Nevertheless, a substantial portion of the dry polymer (>70%) was able to regain its stability, yielding particle sizes ranging from 640 to 900 nm. 10ASR35 outperformed 10ASR15, attributed to its higher concentration of responsive moieties, which facilitated restabilization and release of the primary particles. Despite the better restabilization of 10ASR35, the overall performance depends on the final application. While a higher concentration of MAA is favored for restabilization, it might be disadvantageous in some cases. For instance, when such polymers are used in construction with cement, the complexation of acid groups in the ASR with Ca^2+^ ions can retard the cement hydration process and hinder its setting.^50–52^ A preliminary study of cement hydration in the presence of ASR-based latexes with varying acid contents was conducted and is shown in Section SI.5. Thus, the presence of a higher acid content can be beneficial or detrimental depending on the application.

**Table 8:** Restabilization Features of Kaolin-Reinforced ASR-Based Samples Exposed to Different Conditions

		3 h sediment (%)			
sample original dp	condition	10 min ultraturrax^a^	20 min ultraturrax	30 min ultraturrax	recovered dp (nm) 30 min ultraturrax
10ASR15 530 nm	23 °C	24.4	15.7	12.7	700
	50 °C	29.3	22.6	19.4	750
	50 °C–pressure	35.9	30.7	28.2	900
10ASR35 320 nm	23 °C	16.4	9.5	7.3	640
	50 °C	19.8	14.2	10.8	850
	50 °C–pressure	32.4	26.1	23.4	740

a Stirring at 5000 rpm.

#### 3.3.2 Effect of pH on Restabilization and Hydrolysis

The effect of pH on the restabilization of kaolin-reinforced 10ASR35 was investigated by checking the sedimentation percentage during restabilization at different pH levels, as shown in Figure 9. At pH 4.8, restabilization was poor, with high sedimentation observed. At pH 7.1, a significant improvement occurred due to effective deprotonation of the acid groups, resulting in a lower sedimentation percentage. Increasing the pH to 8.9 and 11.8 further enhanced restabilization, reducing sedimentation to approximately 7%. This behavior is promising as alkaline pH values are often used in applications of polymer latexes as binders, such as in paints and cementitious mixtures. However, elevated pH can induce vinyl acetate hydrolysis, generating alcohol groups and acetic acid.^28^ The hydrolysis degree was monitored by measuring the acetic acid content in the restabilized latexes at pH 8.9 and 11.8 (Figure 9). A higher degree of hydrolysis was observed at pH 11.8 compared to pH 8.9. Despite the high pH employed, the hydrolysis remained very low in all cases due to the hydrolysis resistance provided by VeoVa10.

**Figure 9:**
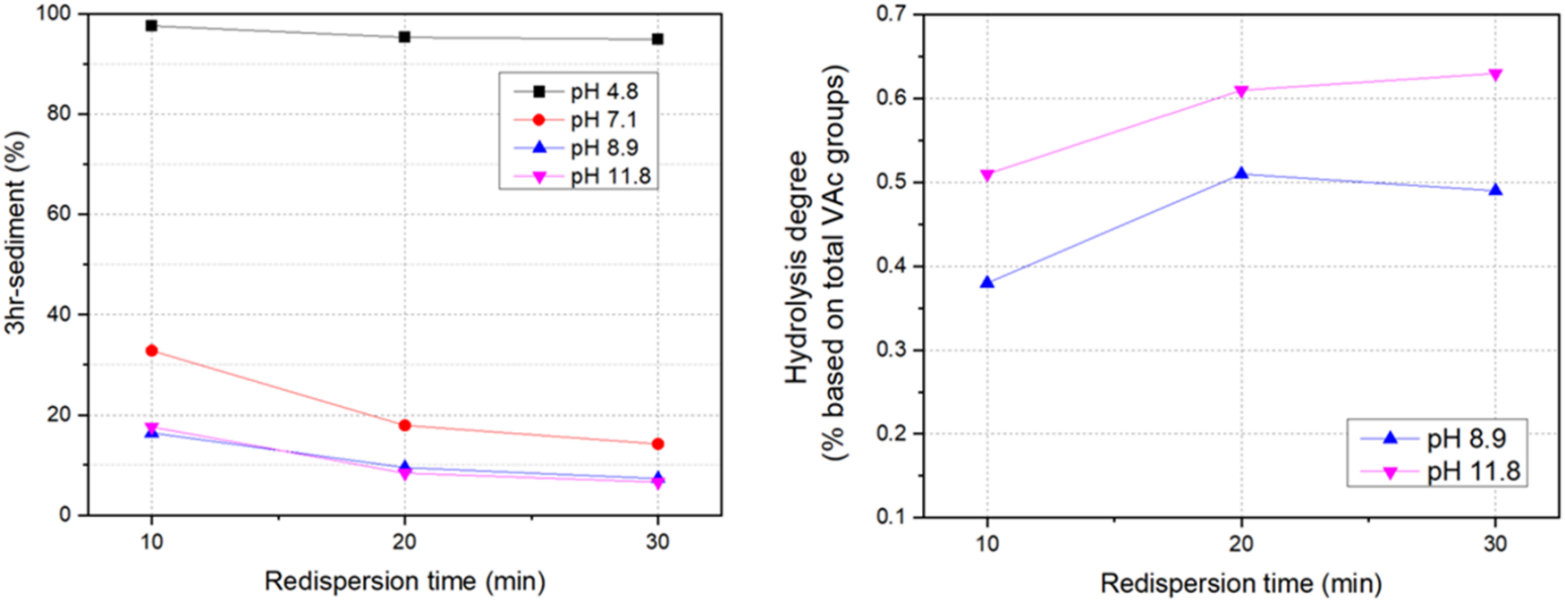
Effect of pH on restabilization (left) and the subsequent degree of hydrolysis (right) of kaolin-reinforced 10ASR35.

## 4 Conclusions

The study successfully synthesized and characterized pH-responsive latexes using alkali-soluble resins (ASRs) as stabilizers in the form of seed for emulsion copolymerization of vinyl acetate (VAc) and vinyl neodecanoate (VeoVa10). ASRs of varying methacrylic acid (MAA) contents including 15, 25, and 35 wt % were employed. High conversions (above 97%) were achieved in all cases, with particle sizes ranging from 320 to 530 nm. The ASRs provided the latexes with good stability and surface incorporation of acid groups, with more than 80% of ASR chains incorporated into the particles. All latexes were successfully coagulated by decreasing the pH to 3, and dry polymer aggregates were obtained by water filtration, drying, and grinding. The obtained aggregates exhibited efficient restabilization in water using Ultraturrax, with submicron recovered particle sizes and low sedimentation level (<10%), demonstrating the potential of ASRs as effective pH-responsive protective colloids.

Restabilization behavior improved by increasing the MAA content in the ASR. Exposing the powders to 50 °C resulted in a blocky material and reduced the restabilization efficiency due to increased particle deformation and the likelihood of sintering. This was effectively addressed by the dry addition of 8 wt % kaolin, which limited the surface contact of polymer aggregates. The direct mixing of kaolin with the latex prior to coagulation was less effective due to the pH sensitivity of kaolin. As an anticaking agent, kaolin preserved the free-flowing behavior of the dry aggregates subjected to temperature and pressure. Restabilization was reduced by applying pressure, although a major portion of the polymer could still be restabilized.

This work demonstrates the feasibility of using ASRs to create switchable, pH-responsive latexes with promising restabilization at alkaline pH, offering uniform polymer distribution for final application as a binder. This is achieved through the effective incorporation of acid groups onto the particle surfaces using ASRs, which proved more efficient than the *in situ* incorporation of acid groups via functional monomers. Additionally, despite the high pH conditions typically required for polymer latex applications, the ASR-based polymers maintained a low degree of hydrolysis due to the incorporation of VeoVa10 units.

## Supplementary Material



## Notes

Description of the techniques used to characterize the polymer latex including conversion, particle size, glass transition temperatures and minimum film formation temperature, molar mass distribution, particle morphology, degree of hydrolysis, and viscosity; thermogravimetric analysis of the latex powder with and without kaolin; *Z*-potential of aqueous kaolin dispersions; and calorimetric analysis of cement hydration in the presence of ASR-based polymers (PDF)
