# Supplementary material for: Alkali-Soluble Resins as pH-Responsive Protective Colloids
Source: ACS Appl Mater Interfaces. 2025 Jul 16;17(30):43750–60. doi: 10.1021/acsami.5c12131 (PMC12314869; doi:10.1021/acsami.5c12131)
Supplement: Supplementary file 1 [file am5c12131_si_001.pdf]

Supporting information for

Alkali Soluble Resins as pH-Responsive Protective Colloids

Mehdi Naderi[1], Kiarash Farajzadehahary[1], Timo Melchin[2], Hans-Peter Weitzel[2], Jose R. Leiza[1*] and José M. Asua[1]

[1]POLYMAT and Department of Applied Chemistry, Faculty of Chemistry, University of the Basque Country UPV/EHU, Joxe Mari Korta Center, Avenida Tolosa 72, 20018, Donostia-San Sebastián, Spain

[2]Wacker Chemie AG, Johannes-Hess-Str. 24, 84489 Burghausen

*Corresponding author, E-mail address: jrleiza@ehu.eus

SI.1. Characterization

The solids content and instantaneous monomer conversion of the latex were determined gravimetrically. Latex samples taken from the reaction were transferred to aluminum pans with the addition of 1–2 drops of a hydroquinone (1 wt%) solution. The samples were then dried in an oven at 65 ˚C overnight. The following equations were employed to calculate the solids content and instantaneous conversion (X_{inst}) at time t:

$$Solids\ content\ (t) = \frac{Weight\ of\ the\ solids(t)}{Weight\ of\ the\ latex\ sample(t)} \qquad (1)$$

$$X_{inst}\ (t) = \frac{Solids\ content\ (t) - Ws(t)}{Wm(t)} \qquad (2)$$

Where Ws(t) is the fraction of initiator, ASR and NaOH in the reactor and Wm(t) is the fraction of fed monomer at the sampling time t.

Particle size distribution (PSD) through dynamic light scattering (DLS) and zeta potential of the dispersions were determined employing a Zetasizer Nano Z (Malvern Instruments). Samples were prepared by diluting a fraction of the dispersion with deionized water. The equipment operated at 25 ˚C, and the reported particle size (dp) and zeta potential values represent the average of three repeated measurements. Additionally, laser diffraction, facilitated by a Malvern Mastersizer, was utilized to analyze the size of the aggregates.

Differential scanning calorimetry (DSC) was employed for the measurement of the glass transition temperature (Tg) of the dried latex polymers using a Q2000 instrument from TA Instruments. The following procedure was followed for characterizations: the sample was initially cooled to -50 ˚C and maintained at this temperature for 1 minute. Subsequently, it was heated to 200 ˚C at a rate of 10 ˚C/min and held at this

temperature for 15 minutes. Following this, it was cooled again to -50 ˚C at a rate of 20 ˚C/min and re-heated to 200 ˚C at a rate of 10 ˚C/min. The second heating cycle was utilized for Tg measurement.

TEM analysis was carried out using a TECNAI G2 20 TWIN operated at 200 kV and equipped with LaB6 filament. The sample was prepared by producing a film from a redispersed dispersion of latex 10ASR35 that was first coagulated and redispersed as explained above. Ultrathin sections of approximately 80 nm thickness were obtained at -25°C using a cryoultramicrotome (Leica EMFC6) equipped with a diamond knife. The sections were placed on 300 mesh copper grids and stained with RuO4 vapor for 20 minutes.

The MFFT measurements were performed using an MFFT-Bar (model 90 from Rhopoint). All the films were cast using a film applicator with 200 µm wet thickness. The MFFT was measured after the sample was dried, and it was defined as the point at which continuous homogeneous films without cracks were observed.

Size exclusion chromatography (SEC-GPC/RI) was employed for the analysis of the molecular weight of ASR polymer samples using an HPLC pump (LC 20AD, Shimadzu) at a flow rate of 1 mL/min. The system comprised three Styragel columns in series (guard column + Styragel HR4, HR2, and HR1, Waters) maintained at 30°C. Detection was performed using a refractive index detector (Waters 2414) also maintained at 30°C. ASR polymer solutions with a concentration of 2 mg/mL were prepared by dissolving the dry polymer in GPC-grade tetrahydrofuran (THF), followed by filtration through a polyamide filter (45 µm pore size) prior to injection into the SEC instrument. The system was calibrated using polystyrene standards ranging from 106-436,200 g/mol.

To investigate the organic/inorganic composition of the initial dry polymer and its sediment after restabilization, thermogravimetric analysis (TGA) was performed on the inorganic fillers, dry kaolin-reinforced polymers, and dried sediments (after 1 day at 50 ˚C), using a Perkin Helmer TGA 8000TM Thermogravimetric Analyzer. Samples weighing 5 – 10 mg were subjected to heating from 40 ˚C to 800 ˚C at a rate of 10 ˚C/min.

The viscosity of the samples collected during the destabilization process was measured using a parallel plate rheometer (TA Instruments, AR1500ex). A steel plate with a diameter of 40 mm and a 2 mm gap between plates was employed. The experiment was conducted at 25 ˚C, applying a constant shear rate of 100 s^{-1}.

The degree of hydrolysis (DH) of the original and restabilized latexes was determined using gas chromatography (GC) to measure the amount of acetic acid in the latex, a by-product of vinyl acetate hydrolysis. GC analysis was conducted with a BP21 column (SGE) on a GC-14A instrument (Shimadzu). Calibration was performed using solutions of various acetic acid concentrations in THF, with 1-pentanol as the standard. Calibration samples included 5 g of solvent (THF), 40 mg of standard (1-pentanol), and different concentrations of acetic acid. Latex samples for injection were prepared by mixing approximately 5 g of diluted latex (10 wt% solids content) with 20 mg of hydroquinone (HQ) aqueous solution (1 wt%) and 40 mg of the external standard. The mixture was stirred magnetically for 1 hour before injection. A 0.5 μl sample was injected using a GC syringe. Prior to the first injection in each set of experiments and after each experiment, a cleaning program was implemented by maintaining the column at 240 ˚C for several minutes to achieve a stable signal without impurity peaks. The experimental conditions and details are provided in Table S1.

Table S1. The conditions and systems used for GC experiments.

**Compound detected**	**Acetic acid**
**Column**	BP21
**Detector type**	Flame ionization detector (FID)
**Standard**	1-pentanol
**Carrier gas**	Helium
**Injector temperature**	170
**Injector pressure (psi)**	7.11
**Detector temperature (˚C)**	250
**Column temperature program**	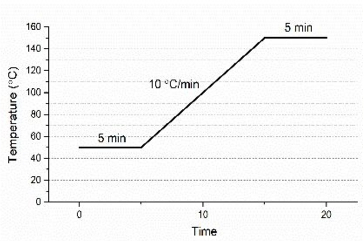

Water-soluble species in the latexes were isolated through centrifugation at 30,000 rpm for 3 hours at 4 ˚C, followed by the collection of the supernatant. A portion of the water phase was dried overnight at room temperature and then subjected to a vacuum oven at 60 ˚C for 1 day. Subsequently, the dry weight was measured, and the water-soluble species content (WSC) based on the total monomers used in the formulation was reported.

SI.2. Characterization of the core-shell morphology

DSC and TEM analysis was performed to further assess that the ASR was incorporated in the surface of the particles even though there was enough indirect evidence that this was the case (e.g., the only stabilization system used was the ASR and stable latexes were obtained, and the coagulated powder was redispersible). Figure S1 shows the DSC traces for an ASR stabilized copolymer and the neat ASR.

a)

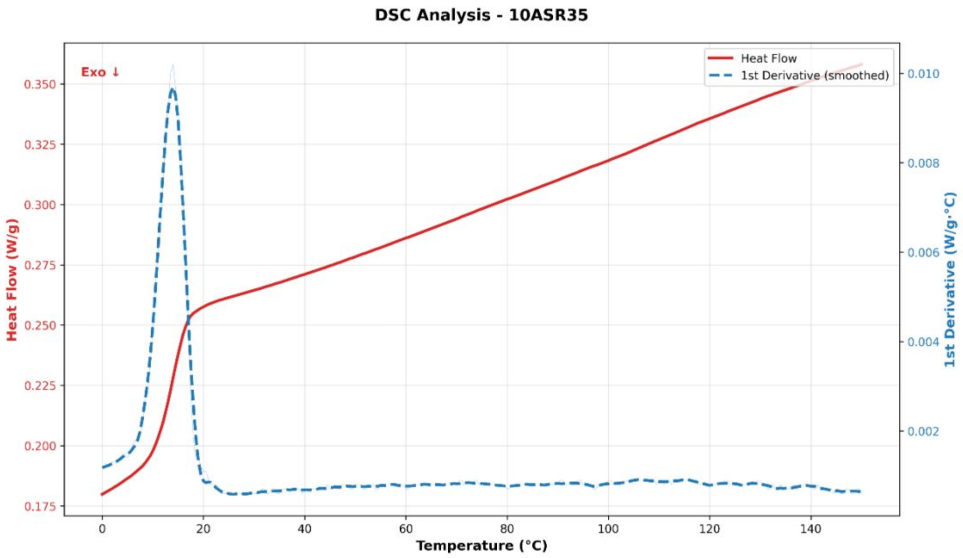

b)

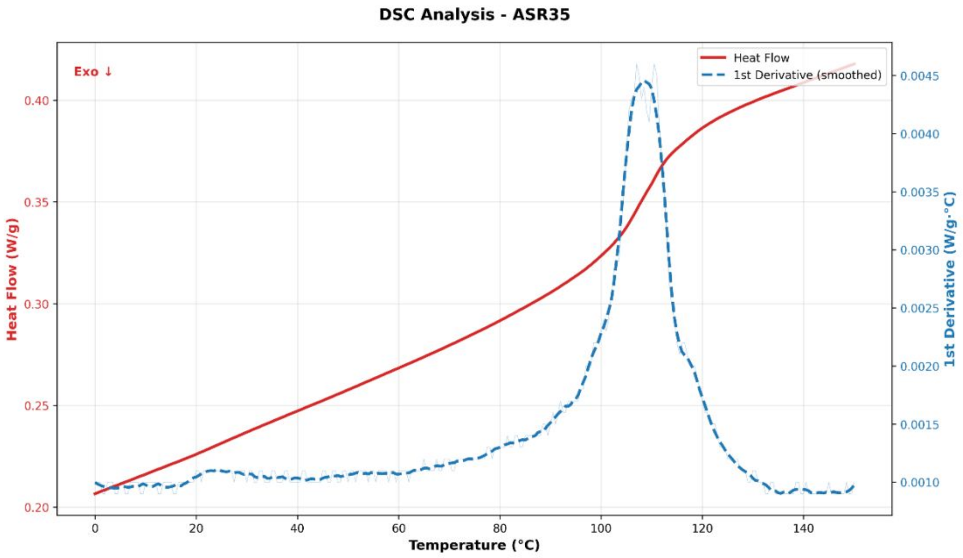

Figure S1. Heat flow and its derivative for measured by DSC for a) the ASR stabilized VAc/VeoVa copolymer, and b) the ASR copolymer.

Figure S2 displays the TEM micrographs at two different magnifications.

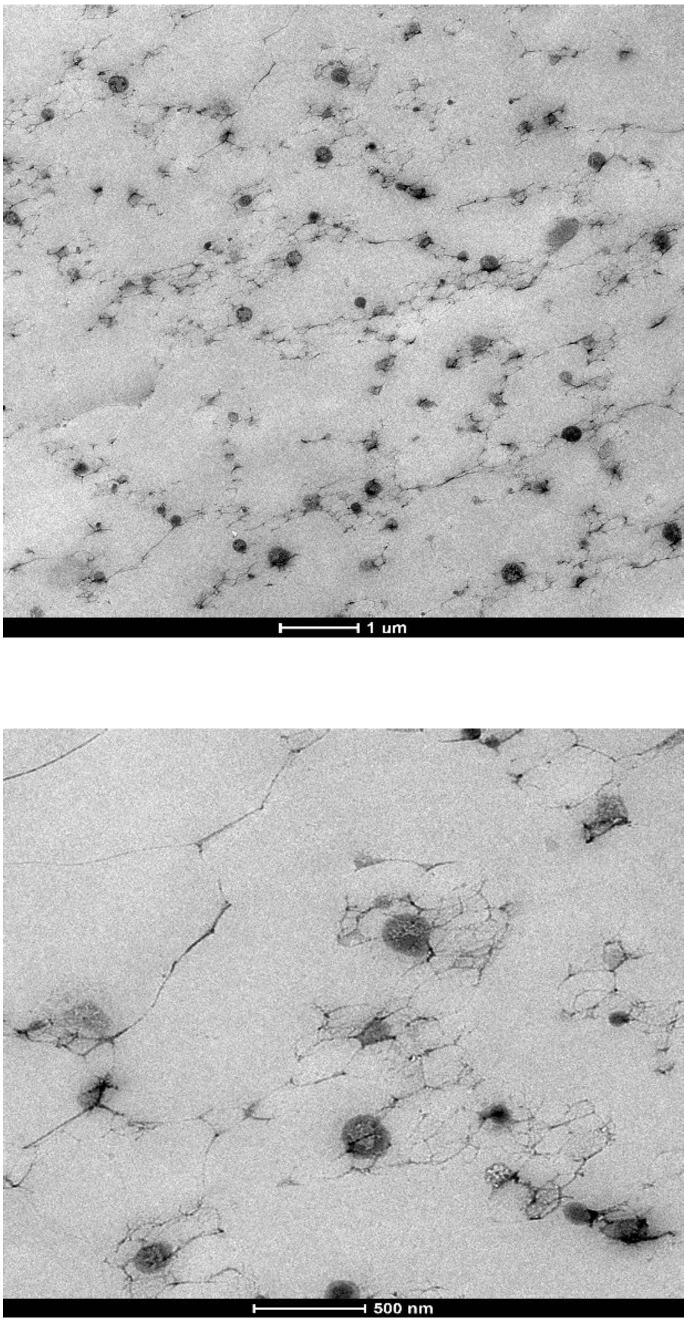

Figure S2. TEM images of a cross-section of a film corresponding to the ASR stabilized copolymer 10ASR35.

SI.3. Thermogravimetric analysis

The dry polymer contains kaolin of high density which can sediment faster than the polymer particles leading to an overestimation in the sediment percentage. To check this, TGA analysis was performed on 10ASR15 exposed to 23 °C for 1 day. The results for the dry 10ASR15, its 3hr-sediment after restabilization as well as its reinforcing filler (kaolin) are shown in Figure S3. There was a significant decline in weight between 300 and 350 ˚C due to the polymer decomposition. After that, the weight decreased more slowly, and after 400 ˚C, there was another fast decline in weight due to kaolin decomposition. The residual amount at 800 ˚C was higher for the sediment than the initial dry polymer, confirming that the sediment contained a higher content of inorganic material as they sediment faster. Considering the residual percentage of pristine kaolin and assuming that the polymer was almost fully decomposed until 800 ˚C, the percentage of inorganic fillers in the dry product and sediment was estimated and reported (Table S2). Comparing with the initial sample, the amount of kaolin increased threefold in the sediment.

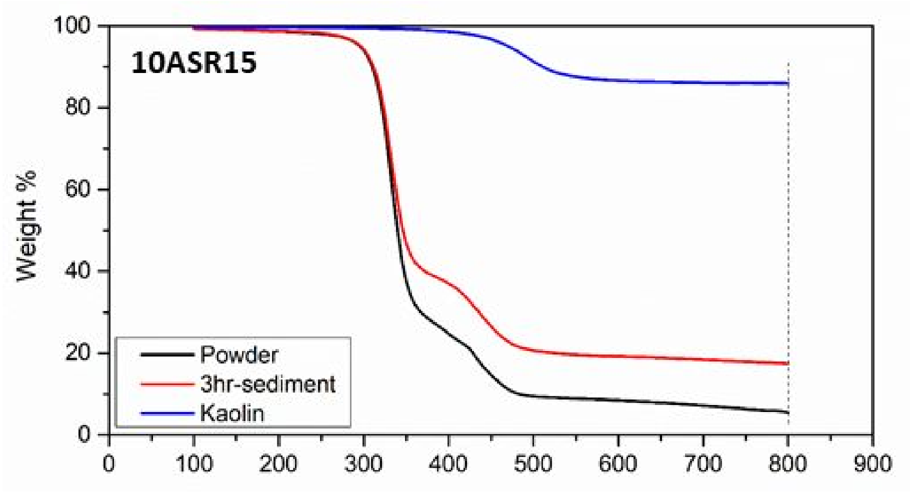

Figure S3. TGA analysis of the reinforced 10ASR15 dry polymer.

Table S2. Organic/inorganic composition of the initial dry sample and its sediments after restabilization.

**Sample**		**Polymer fraction (wt%)**	**Filler fraction (wt%)**
**10ASR15**	Initial dry product	92.59	7.41
	Sediment	79.73	20.27

SI.4. Zeta potential and stability of kaolin dispersion

To comprehend the surprising difference in performance between the kaolin dry and wet mixing strategies, the stability of the kaolin dispersion at various pH levels was investigated. A dispersion containing 1 wt% kaolin in water was created by mixing kaolin with water using an Ultraturrax at 5000 rpm for 10 minutes. The resulting dispersion had a pH of 6.3. Kaolin dispersions with different pH values were prepared using HCl (2 wt%) and NaOH (5 wt%) aqueous solutions. The zeta potential of the dispersions (using a Zetasizer equioment), along with their physical appearance after 3 hours of sedimentation were determined. Figure S4 illustrates that the kaolin dispersion exhibited pH responsiveness, with its zeta potential changing with pH due to the pH responsiveness of kaolin. where the absolute zeta potential value decreases with a reduction in pH, impacting the stability of the dispersion. At pH levels of 6.3 and 10, high absolute values of zeta potential were observed, indicating stability of the kaolin particles, and the dispersion remained turbid after 3 hours. Conversely, at pH levels of 2 and 3.5, lower absolute values of zeta potential were measured, leading to agglomeration and sedimentation of particles over time.

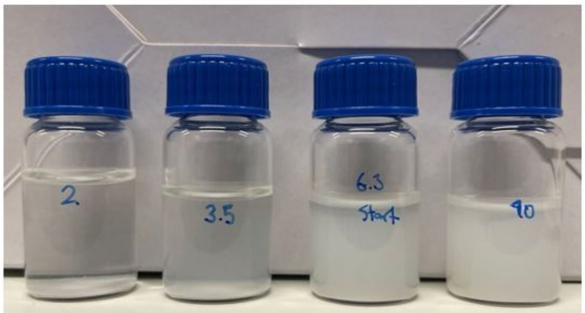

**pH:**	**2**	**3.5**	**6.3**	**10**
**Zeta potential:**	**3**	**-18**	**-45**	**-53**

Figure S4. Stability and zeta potential of kaolin dispersion at different pHs.

SI.5. Effect of ASR-based latexes on cement hydration

Calorimetry results of cement (OPC, CEM Type I 52.5R) in the presence of ASR-based polymers with different acid contents, were examined (Figure S5). For 10ASR15, the hydration peak was observed after a few hours. The high concentration of acid groups in 10ASR35 polymer significantly hindered hydration, making it unsuitable for mortar applications. The test procedure was conducted through isothermal calorimetry using TAM Air instruments from TA Instruments. The direct addition method was employed, involving the addition of the polymer dispersion to the dry cement powder in the test vial. This was followed by 90 seconds of mixing using a vortex mixer, a 60-second rest, and another 90 seconds of mixing. Subsequently, the vial was promptly placed in the equipment right after the mixing process. A total mass of

5 grams was used, maintaining a water/cement ratio of 0.4 with varying polymer/cement ratios. A reference paste comprising only cement and water was prepared for comparative analysis.

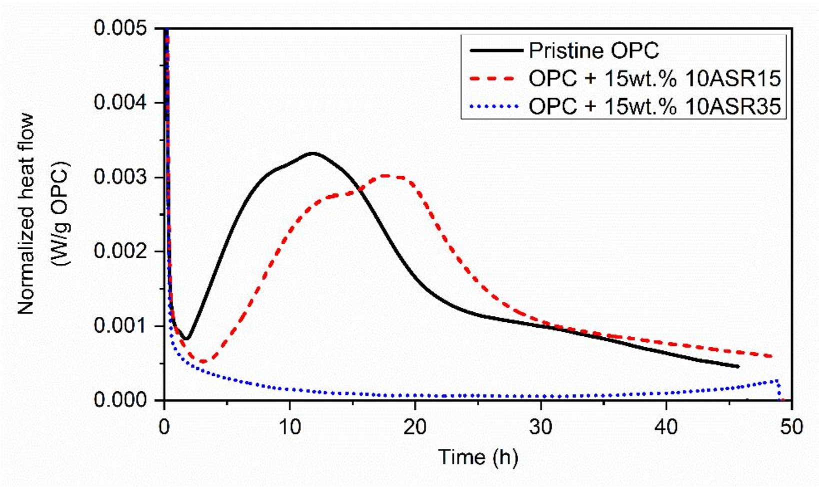

Figure S5. Cement calorimetry in presence of 15 wt% ASR-based polymers of different acid content.
